# Parents’ Perceptions: Environments and the Contextual Strategies of Parents to Support the Participation of Children and Adolescents with Autism Spectrum Disorder—A Descriptive Population-Based Study from Switzerland

**DOI:** 10.1007/s10803-022-05826-2

**Published:** 2022-12-20

**Authors:** Beate Krieger, Albine Moser, Thomas Morgenthaler, Anna J. H. M. Beurskens, Barbara Piškur

**Affiliations:** 1 School of Health Sciences, ZHAW Zurich University of Applied Sciences, Katharina Sulzer Platz 9, 8401 Winterthur, Switzerland; 2 Department of Family Medicine, School Caphri, Maastricht University, Maastricht, The Netherlands; 3 Research Centre for Autonomy and Participation for People With Chronic Illness, Zuyd University of Applied Sciences, Heerlen, The Netherlands

**Keywords:** Autism, Youth, Context, Home, School, Community, Inclusion, PEM-CY

## Abstract

Environments have a modifying effect on the participation of children and adolescents with autism spectrum disorder (ASD) in all areas of life. This cross-sectional study investigated parental perspectives on supportive or hindering environments and the daily contextual strategies parents used to enhance their children’s participation. Qualitative and quantitative data gathered from 115 parents from German-speaking Switzerland using the participation and environment measure-child and youth (PEM-CY) were analyzed. Results revealed 45 environmental supports and barriers at home, at school, and in the community. Contextual strategies were identified in combination with people, activities, time, objects, and places. Parental perspectives on participation and their contextual strategies should be considered in environmental-based interventions to support the participation of children and adolescents with ASD.

**Supplementary Information:**

The online version contains supplementary material available at 10.1007/s10803-022-05826-2.

## Introduction

The environment can be a support or a barrier to the participation of children and adolescents with autism spectrum disorder (ASD*)* in all areas of life and it plays an important role in their development, health, and wellbeing (Askari et al., 2015)*.* For example, sensory responsiveness to physical features such as noise or light has been described as a barrier to their participation (Gabriels et al., 2008), while enacted friendships can protect against anxiety and loneliness in children and adolescents with ASD and thus can serve as a supportive environment (Lasgaard et al., 2010). The World Health Organization’s (WHO) definition of participation as “*involvement in life situations”* (WHO, 2001, 2007) is extended here to include *“being engaged in and/or performing meaningful activities in occupational and social roles while attending”* (Krieger et al., 2018, p. 2). Activities like after-school sports, shared family activities, or doing homework (Obrusnikova & Cavalier, 2011; Orsmond et al., 2006; Zaidman-Zait et al., 2014) are embedded in an environment, which plays a mediating role in the participation of children with disabilities (Anaby et al., 2014; Askari et al., 2015; Myers et al., 2015; Shattuck et al., 2011; Sood et al., 2014). To explore the multi-dimensional construct of the environment, this article differentiates between environment, setting, and context (see Table 1), which all encompass external conditions that affect participation: environments most generally, and context most specifically.

**Table 1:** Definitions

Participation	The definition of WHO “*involvement in life situations”* (WHO, 2001) is extended here to include *“being engaged in and/or performing meaningful activities in occupational and social roles while attending”* (Krieger et al., 2018, p. 2)
Environment	*“The physical, social, and attitudinal environment in which people live and conduct their lives”* (WHO, 2007, p. 5). The environment can be a support or a barrier for participation
Setting	Used in PEM-CY to cluster a group of contexts with similar circumstances and conditions such as “home,””school,” and “community.”
Context	*“Experienced and situated activity settings” *(King et al., 2018, p. 1835) Contexts are described with five inherent elements: people, place, activity, objects, and time. Contexts are where transactions take place and their effects can be noticed

Reduced participation of children and adolescents with ASD has been reported in settings such as home, school, or the community. At home, they participate less frequently and are less involved in personal care activities and socializing with other people (Egilson et al., 2018; Little et al., 2014). At school, less frequent socializing activities, fewer friends, and less frequent physical activities are reported (Symes & Humphrey, 2010; Wainscot et al., 2008). In the community, their participation in leisure activities, socializing with peers, attending public events, or using public services is reduced as well (Egilson et al., 2017; Hilton et al., 2008; Obrusnikova & Miccinello, 2012; Shattuck et al., 2011).

Concerning the environment, there is emerging evidence that environments as seen from parents’ perspectives are less supportive of the participation of children and adolescents with ASD compared to their peers without disabilities (Devenish et al., 2020; Egilson et al., 2017; Lamash et al., 2019). Because environment and participation are inherently associated with national socio-economical factors, previous research from Israel (Lamash et al., 2019) and Iceland (Egilson et al., 2017) needs to be supplemented with additional national data.

Qualitative research from Switzerland has explored environmental supports and barriers to the participation of adolescents with ASD and found that adolescents are dependent on environmental pre-requisites to even attend participation (). Parent advocate groups in the field of autism aim to implement autistic-friendly environments in malls and cinemas (Autismus Deutsche Schweiz, 2019). However, there is an officially recognized paucity of research describing the living situations of children and adolescents with ASD and their families in generally and specifically with an environmental focus in Switzerland (Bundesrat, 2018).

Family and specifically parents live in a strong “transactional relationship” with their children and adolescents with ASD. The “transactional relationship” between a person and a context (see Table 1) results in changes to both the individual and the environment over time (Hammel et al., 2008; Imms et al., 2017; Kramer et al., 2012; Mallinson & Hammel, 2010; Schneidert et al., 2009). Parents are not only the best-informed people regarding their children and adolescents with ASD but actively create immediate social and physical contexts for their children at home and greatly influence further contexts at school and in the community. The parental role in providing a secure environment and helping to connect children socially has been found to be essential to supporting the participation of adolescents with ASD (Krieger et al., 2018). This contextual support is provided by parental strategies such as planning, motivating, and guiding. Research describes parental strategies for children with physical disabilities (Piškur et al., 2012) but little is known about parental strategies for children with and without ASD in the home setting (Egilson et al., 2018), and to our knowledge, there is no information about school and community settings. Further, strategies tailored specifically to children and adolescents within the diverse spectrum of ASD might differ from those of a general population. In adolescents with ASD and anxiety, reported parental strategies to increase community participation included preparing, practicing participation in advance, and avoiding specific triggers or sensory overload (Adams et al., 2019).

From a societal standpoint, there is a need to understand environmental aspects and parental contextual strategies that foster participation at home, at school, and in the community. This is relevant because identified supports or barriers can be more specifically targeted. Therefore, the purpose of this study is to explore the environmental and contextual aspects affecting the participation of children (age 5 to 11 years) and adolescents (age 12 to 17 years) with ASD in Switzerland from the parental perspective. More specifically, two questions are formulated:

1. What aspects of the environment at home, at school, and in the community do parents of children and adolescents with ASD living in Switzerland describe as supports and as barriers for the participation of their children?
2. What contextual strategies do parents of children and adolescents with ASD describe using to enhance the participation of their children at home, at school, and in the community in Switzerland?

Following Lyon and collegues’s understanding of nomothetic and ideograph reseach aspects (Lyon et al., 2017), this study tries to elaborate with the first question mainly a general statement that account for a larger social pattern that was never described before (defined as normothetic) and with the second question mainly to uncover detailed information about a narrower subject of study (defined as ideographic) to develop a more comprehensive understanding of the role of environments for the participation of children and adolescents with ASD.

## Method

### Design

A population-based cross-sectional descriptive study was selected, describing features at a given point in time. ‘Environment,’ defined according to the definition of the World Health Organization (see Table 1), can be qualified as barriers or as supports to participation and are addessed with research question 1. Supporting environments are those addressing the human desires to explore, understand, enhance competence, be part of the solution, and participate with others towards meaningful goals (Kaplan & Kaplan, 2008, 2009). Parental contextual strategies are addressed with research question 2. They are enacted in “experienced and situated activity settings” (King et al., 2018; p. 1835) and are described with five inherent elements of contexts: people, place, activity, objects, and time (King et al., 2018).

### Participants

Participants were German-speaking parents who care for one or more children between 5 and 17 years with a recognized medical diagnosis of ASD according to ICD-10 (actual diagnostic classification in Switzerland). Excluded were parents who do not live together with their children with ASD. For the self-selected sample, parents were recruited through invitation letters sent to multiple different pathways in the German-speaking part of Switzerland, such as client and professional organizations, social media and webpages, and specialized medical services and schools for children with ASD. The invitation letter included a link to a webpage with further information and an online survey. Participation was voluntary and the given information was anonymous in accordance with the data safety laws of the European Community. Parents consented online with informed consent. This survey received a jurisdictional declaration of non-objection by the cantonal ethical committee of Zurich (BASEC Request 2018-00238).

### Measures

The online questionnaire consisted of the German version of the participation and environment measure-child and youth (PEM-CY(G) (Coster et al., 2012; Krieger et al., 2020b), demographic questions, and questions about the actual manifestations of ASD symptoms.

The demographic questions included the family constellation, living in urban, suburban or rural communities, education of parents and the actual percentage of paid work. This indicates the socioeconomic status of the family. The research team refrained to ask for the annual income and marital status, as this declaration is not common to Switzerland. Cultural diversity was reflected on the reported languages spoken in each family. It was estimated that clinical diagnostic features were either unknown to parents or not currently up to date. In Switzerland, until now the ICD-10 criteria are mostly used for ASD diagnostic. Therefore, we developed an actual ASD-manifestations questionnaire according to ICD-10 diagnostic criteria (e.g. communication, restricted and repetitive behavior) and reported challenges from parents of children with ASD (e.g. ability to express themselves, difficulties handling change, sleeping situation, self-injuring behavior, age-appropriate independence) (Galpin et al., 2018). The eleven manifestations were rated for the last 4 months on a Likert-type scale between 1 and 6. The scales were formulated with qualitative anchors (e.g., 6 = “Our child can express himself or herself age-appropriately or better” versus 1 = “The expressive language of our child is very low”). The questionnaire was scrutinized by three clinical ASD experts for completeness and readability; consequentially, minor amendments were made.

The PEM-CY is a standardized parent-reported assessment of the extent and patterns of participation of children and youth between 5 and 17 years and parents’ desires for change in three different settings (home, school, and community). For each of these three settings, 10 to 17 different environmental aspects, divided into “helpfulness” (e.g., sensory features, demands, socializing persons, attitudes) and “resources” (e.g., services, information, time, or money) are assessed. Parents are asked whether these aspects are helpful or available, which are operationalized in PEM-CY as “supports,” or not helpful, which are operationalized in PEM-CY as “barriers.” These were used to answer the first research question. To answer the second research question, parents are further asked in the PEM-CY (G) with open-ended questions to describe three contextual strategies that they apply to support the participation of their children in each setting. PEM-CY has demonstrated adequate internal consistency and test–retest reliability (Coster et al., 2011). It has been applied to young people with ASD (Devenish et al., 2020; Egilson et al., 2017, 2018; Lamash et al., 2019; Simpson et al., 2018; Tint et al., 2017; Weiss & Burnham Riosa, 2015). For this study, a German-translated and cross-cultural adapted version of the PEM-CY(G) (Krieger et al., 2020b) was used.

The questionnaire was set up on an online platform (SocSicSurvey.com). For piloting, five parents of children with ASD provided feedback on comprehensiveness and user-friendliness and it was changed accordingly. As data collection took place between the 6 weeks of Covid-19 lockdown and restrictions, parents were asked to refer to participation experiences in the time before the lockdown for their answers.

### Analysis

The data were analyzed using IBM SPSS Statistics (Version 27.1) analytical software.

### Demographics

Demographics of parents and youth were summarized and total numbers, percentages, and means and/or medians were calculated for the whole sample and two age groups (children: 5–11 years and adolescents: 12–17 years). This age division reflects the difference between primary and secondary school in Switzerland. Based on the data level, *t*-tests or Mann–Whitney *U* tests were performed to indicate a statistically significant difference level of *p* = 0.05 for demographic data. Parent-reported manifestation of ASD was calculated using the median and quartile range for the whole group and the two sub-groups. Calculations were based on Tukey’s range tests to account for the ordinal scale used.

### Analysis of Environmental “Supports” and “Barriers”(Research Question 1)

PEM-CY measures 45 total environmental aspects. Their number varies per setting (home: 12; school: 17; community: 16). As recommended by the PEM-CY user guide, environmental aspects judged with *“usually helps”* and *“usually yes”* were viewed as “supports” and those judged with *“usually makes harder”* or *“usually no”* were viewed as “barriers.” We calculated the average amount of perceived “support” and “barriers” for all three settings, both age groups, and the whole sample. For more in-depth insight, for each of the 45 environmental aspects the percentage of parents who opted for a particular answer in each age group and in the whole sample was calculated.

### Analysis of Parental Contextual Strategies to Support Participation (Research Question 2)

For each setting (home, school, and community), parents were asked in an open-ended question (part of the PEM-CY questionnaire) to list three strategies that they use to support their children’s participation. Due to received rich data, the research team decided on a secondary analysis by posing another research question beyond those originally intended with the primary data (Ruggiano & Perry, 2019). The same two researchers who performed a first analysis (BK, TM) applied a summative content analysis, typically used to explore word usage or contents in texts (Hsieh & Shannon, 2005). It is seen as a deductive approach (Armat et al., 2018). The two native German-speaking researchers used the five aspects of context (people, place, activity, objects, and time) as defined by King et al. (2018) as pre-determined codes. After agreeing on definitions in a codebook, both coded the written comments of parents, which were separated for the three settings (home, school, and community) and were divided into two age groups (children and adolescents). Next, they compared, discussed, and reflected on their results. After saturation was reached, which means that no new codes or themes emerged, they presented the qualitative results to a third native-speaking researcher (AM) to refine it further. After translating the results and exemplary codes into English, they presented the results to the whole research team until saturation on positioning of all codes was reached. Finally, they agreed on calculations, contents of the table, and a narrative summary report. To ensure trustworthiness (Guba & Lincoln, 1985), all received written strategies were coded and data triangulation with quantitative and qualitative aspects ensured credibility. Researcher triangulation contributed to the confirmability of the study. Transferability was guaranteed by providing a well-documented context and research process.

## Results

Results are divided into three parts: (1) the description of the study sample; (2) the presentation of parents’ perceptions of environmental aspects as “supports” and “barriers” in 45 environmental aspects along the three settings differentiated between the two age groups (research question 1); (3) and a summary of the analysis of 623 received comments about the contextual strategies of parents according to the five aspects of context: people, place, activity, objects, and time (reseach question 2).

### Description of the Study Sample

The final analysis included 115 participants. The flow of eligibility is additionally given as supplementary file (see Online Resource1). The demographics of the 115 parents are listed in Table 2. Overall, 60 parents reported on children (5–11 years of age) while 55 reported on adolescents (12–17 years of age). Statistically, the two groups did not differ significantly *(p* = 0.05) with regards to responding parents, community type of residence, and education level of parents. However, the family constellation differed statistically in adolescents, who lived more often in separated households. Further, the paid working hours of mothers of adolescents with ASD were higher than those of mothers of children with ASD.

**Table 2:** Demographic characteristics of parents answering questions for their children or adolescents with ASD

	Children with ASD		Adolescents with ASD		Total group of youth	
	Age 5–11, *N* = 60		Age 12–17, *N* = 55		Age 5–17, *N* = 115	
	*N*	%	*N*	%	*N*	%
Responding persons						
Mother^a^	46	78.0	49	89.1	95	83.3
Father^a^	9	15.3	4	7.3	13	11.4
Both together^a^	4	6.8	2	3.6	6	5.3
Community type of living						
Urban^a^	9	15.0	5	9.1	14	12.2
Rural^a^	38	63.3	28	50.9	66	57.4
Agglomeration (suburbs)	13	21.7	22	40.0	35	30.4
Family constellation						
Child lives with both parents together	52	86.7	41	74.5	93	80.9
Parents separated; child lives in two households	0	0.0	4	7.3	4	3.5
Parents separated; child lives overly with one parent^a^	5	8.3	6	10.9	11	9.6
Child lives with one parent in a new family^a^	2	3.3	2	3.6	4	3.5
Other or missing^a^	1	1.7	2	3.6	3	2.6
Number of siblings of child with ASD						
No siblings	22	36.7	13	24.1	35	30.7
One sibling^a^	24	40.0	26	48.1	50	43.9
Two or more siblings^a^	14	23.3	15	21.7	29	25.5
Education of mother						
Obligatory^a^	3	5.0	2	2.6	5	4.3
Secondary education^a^	17	28.4	18	32.7	35	30.4
Tertiary education^a^	40	66.7	33	63.6	75	65.3
Unknown or missing^a^	0	0.0	0	0.0	0	0.0
Education of father						
Obligatory^a^	1	1.7	1	1.8	2	1,7
Secondary education^a^	15	25.0	11	20.0	26	22.6
Tertiary education^a^	42	70.0	39	71.0	81	70.4
Unknown or missing	2	3.3	4	7.2	6	5.3
Further information of the family						
Actual percentage of paid work of mother	33.1^b^	31.32^c^	49.9^2^	33.25^c^	41.1^b^	33.20^c^
Actual percentage of paid work of father	87.1^b^	27.31^c^	80.5^2^	33.76^c^	84.0^b^	30.57^c^
Number of languages spoken in the inner family	1.42^b^	0.72^c^	1.53^2^	1.42^c^	1.47^b^	1.1^c^

^a^No significant difference at the *p* = 0.05 level

^b^Mean

^c^SD

The demographics of the children and adolescents with ASD are listed in Table 3. The two age groups differed statistically in the average age of diagnosis, number diagnosed with Asperger’s syndrome, and the number of friends and peers (meetings via social media) they meet with per week, whereas 15–20% of parental answers for informal friendships and social media contacts were missing.

**Table 3:** Demographic characteristics of children and adolescents with ASD as described by parents

	Children with ASD		Adolescents with ASD		Total group of youth	
	Age 5–11 y; *N* = 60		Age 12–17 y; *N* = 55		Age 5–17 y; *N* = 115	
	*N*	%	*N*	%	*N*	%
Gender						
Male^a^	51	85.0	41	74.5	92	80.0
Female^a^	9	15.0	14	25.5	23	20.0
Type of ASD						
Autism spectrum disorder^a^	16	26.7	11	20.0	27	23.5
Early onset autism^a^	10	16.7	8	14.5	18	15.7
Asperger Syndrome	23	38.3	33	60.0	56	48.7
Atypical autism^a^	5	8.3	3	5.5	8	7.0
Other not specified	6	10.0	0	0.0	6	5.2
Age of diagnosis	5.6^2^	2.12^3^	8.5^2^	3.41^3^	7.0^2^	3.15^3^
Co-occuring diagnosis						
No co-occuring diagnosis	34	56.7	19	34.5	53	46.1
ADHD^a^	11	18.3	12	21.8	23	20.0
Anxiety^a^	1	1.7	4	7.3	5	4.3
Epilepsy^a^	0	0.0	1	1.8	1	0.9
Depression^a^	1	1.7	4	7.3	5	4.3
Motor dysfunction^a^	4	6.7	2	3.6	6	5.2
Others or unknown	6	10.0	11	20.0	14	14.8
Schooling						
Regular setting without adjustments^a^	8	13.6	8	14.8	16	14.2
Regular setting (minor adjustments)^a^	20	33.9	13	24.1	33	29.9
Regular setting (special adjustments)^a^	9	15.3	6	11.1	15	13.3
Private school^a^	7	11.9	13	24.1	20	17.7
General separate school^a^	13	22.0	8	14.8	21	18.6
Home or boarding school^a^	1	1.7	2	3.8	3	2.7
Vocational apprenticeship	0	0.0	3	5.6	3	2.7
None or misssing^a^	1	1.7	3	5.6	4	3.6
Friendships (outside school)						
Personal friends meeting per week	1.4^b^	2.04^c^	0.7^b^	0.94^c^	1.1	1.64^c^
Informal peers meeting per week^a^	2.4^b^	4.64^c^	3.0	4.22^c^	2.8	4.41^c^
Peers meeting via social media per week	0.6^b^	1.81^c^	3.1	4.33^c^	1.8	3.55^c^

^a^No significant difference at the *p* = 0.05 level

^b^Mean

^c^SD

The distribution of autistic characteristics in the children and adolescents with ASD was determined from the statements of the parents (see supplementary file Online Resource 2) *“Expressive language ability,*” “*use of communication aids,” “intellectual abilities,”* and “*no self-harming behavior*” were rated highly in both groups, indicating a less severely challenged autistic sample. “*Reaction to change,”* “*repetitive behavior,” “restricted behavior,”* and “*selective eating pattern,”* were rated in the middle range for both sub-groups. In contrast, *“sleeping situation,”* “*interaction with other children,”* and “*age-appropriate independence”* scored in the middle range, though there was more variation in adolescents with ASD than in children with ASD. Solely *“reaction to change”* presented with a high spread in both groups. Overall this is read as a sample with overall “high functioning ASD”.

### Parents’ Perceptions of Environmental Aspects in Three Settings

Table 4 lists the percentages of parents’ perceived judgements of the 45 environmental aspects in three settings. Half of all parents indicated clear environmental “supports” and “barriers” to the participation of their children. From this half, both age groups were perceived to have more “supports” than “barriers” in all three settings. The average number of perceived supports in the three settings (home: *M* = 4.6, SD = 2.3; school: *M* = 6.2, SD = 3.4; community *M* = 5.4, SD = 2.7) was over 50% higher than the average number of perceived “barriers” (home: *M* = 1.1, SD = 1.3; school: *M* = 2.8, SD = 3.0; community *M* = 3.0, SD = 3.0). There were only minor differences between the age groups.

**Table 4:** List of 45 environmental aspects in three settings: percentages of parents

			*N*				Supports						Barriers		
				All	< 11	> 11	All	< 11	> 11	All	< 11	> 11	All	< 11	> 11
Home environment	Helpfulness	*"Do the following things help or make it harder to participate in activities at home?"*		"Not an issue"			"Usually helps"			"Sometimes helps, sometimes makes harder"			"Usually makes harder"		
		1. Physical layout	113	35	13	21	24	14	10	36	21	15	5	3	3
		2. Sensory quality	113	19	10	10	15	7	8	**42**	20	22	23	14	9
		3. Physical demands of activity	114	35	16	13	16	8	8	39	21	18	16	7	9
		4. Cognitive demands of activity	113	27	12	15	21	12	9	37	21	16	15	6	9
		5. Social demands of activity	113	16	7	9	27	14	12	**40**	23	17	18	7	11
		6. Relations with family members	114	16	8	8	32	18	15	**46**	25	22	5	2	4
		7. Attitudes	106	33	16	17	28	17	11	39	10	19	9	7	3
	Resources	*"Are the following available and/or adequate to support your child’s participation at home?"*		"Not needed"			"Usually yes"			"Sometimes yes, sometimes no"			"Usually not"		
		8. Services	115	**76**	37	39	10	7	3	7	4	3	8	4	3
		9. Supplies	115				**90**	47	43	10	5	4	8	4	3
		10. Information	115				**76**	41	37	20	10	10	2	1	1
		11. Time	115				**52**	25	26	**47**	27	20	2	0	2
		12. Money	115				**65**	33	35	23	16	8	9	3	5
School environment	Helpfulness	*"Do the following things help or make it harder to participate in activities at school?"*		"Not an issue"			"Usually helps"			"Sometimes helps, sometimes makes harder"			"Usually makes harder"		
		1. Physical layout	111	21	8	13	27	15	12	36	17	19	16	10	6
		2. Sensory quality	111	4	0	4	14	7	7	29	17	12	**53**	26	27
		3. Weather conditions	111	**42**	18	24	8	5	4	34	14	20	15	14	2
		4. Physical demands of activity	111	16	4	13	14	8	5	37	20	17	33	19	14
		5. Cognitive demands of activity	110	13	4	9	20	11	9	35	19	15	33	16	16
		6. Social demands of activity	110	6	3	4	12	6	5	**44**	21	23	**50**	20	18
		7. Attitudes	108	5	1	4	**44**	28	17	**40**	17	23	11	6	6
		8. Relations with peers	110	10	5	5	22	14	8	**48**	23	25	20	8	12
		9. Safety	109	22	8	14	37	18	18	24	16	8	17	7	10
	Resources	*"Are the following available and/or adequate to support your child’s participation at school?"*		"Not needed"			"Usually yes"			"Sometimes yes, sometimes no"			"Usually not"		
		10. Personal transportation	112	**63**	32	30	34	18	16	4	1	3	0	0	0
		11. Public transportation	112	**60**	37	23	37	13	24	1	0	1	3	1	2
		12.Programs and services	112	14	6	8	**49**	26	23	19	8	11	18	11	7
		13. Policies and procedures	111	22	9	13	**43**	20	23	20	10	10	15	12	4
		14. Supplies	112				**77**	38	39	19	12	7	4	2	3
		15. Information	111				**63**	32	32	30	15	14	7	4	4
		16. Time	113				**67**	35	32	31	16	15	2	0	2
		17. Money	112				**75**	35	40	19	12	7	6	4	2
Community environment	Helpfulness	*"Do the following things help or make it harder to participate in activities at school?"*		"Not an issue"			"Usually helps"			"Sometimes helps, sometimes makes harder"			"Usually makes harder"		
		1. Physical layout	114	47	20	27	16	11	5	27	15	12	10	6	4
		2. Sensory quality	114	9	2	7	8	4	4	39	20	18	**45**	26	20
		3. Physical demands of activity	113	24	8	16	9	2	7	35	24	11	33	19	14
		4. Cognitive demands of activity	114	30	9	21	7	4	4	29	18	11	34	21	13
		5 Social demands of activity	113	11	4	7	9	5	4	**40**	23	17	**40**	20	20
		6. Relations with peers	113	15	3	9	13	7	6	**42**	24	18	31	15	16
		7. Attitudes	113	7	3	4	19	11	9	**48**	25	23	26	13	13
		8. Weather conditions	114	**45**	19	25	4	2	2	**40**	21	19	11	10	2
		9. Safety	114	18	9	9	32	16	17	28	17	11	22	11	10
	Resources	*"Are the following available and/or adequate to support your child’s participation in the community?"*		"Not needed"			"Usually yes"			"Sometimes yes, sometimes no"			"Usually not"		
		10. Personal transportation	115	17	8	10	**76**	39	37	5	4	1	2	1	1
		11. Public transportation	115	29	18	10	**68**	31	37	3	3	0	1	0	1
		12. Programs and services	115	30	16	14	37	17	20	18	9	10	15	10	5
		13. Information	112				**55**	25	30	31	18	13	13	8	5
		14. Equipment and supplies	112				**65**	30	35	28	18	10	7	3	4
		15.Time	111				**57**	31	26	29	20	18	4	1	3
		16. Money	113				**70**	34	36	22	13	9	8	4	4

Bolded: if more than 40% of all parents opted for this

The other half of parents did not indicate environmental “supports” or “barriers” clearly. Out of these, two groups could be distinguished: first, a third of all parents choose “*sometimes yes/helpful, sometimes no/hard*” (home: 31%; school: 27%; community: 30%), indicating a swing between the environment being a “support” or being a “barrier.” Second, for 20% of all parents, environmental aspects were “*not an issue*” (home: 21%; school: 17%; community:17%). Most of the 115 parents answered all 45 questions. *“Attitudes”* had the lowest answer rate with 108, as seen in Table 5.

**Table 5:** Parental strategies to support participation

Clusters of strategies	Single strategies	Explanation of parental contextual strategies	Three settings (H) (S) (C)			Children (age 5–11 years) exemplary quotes from home (H), school (S), and community(C)	Adolescents (age 12–17 years) exemplary quotes from home (H), school (S), and community(C)
*Theme: “PEOPLE”*: parents use their relationship to support participation (257 comments: 130 children; 127 adolescents)							
Encouraging participation (62 comments)	Confirming and motivating (46 comments)	Parents try to motivate. They give positive feedback and encourage involvement	x	x	x	“Confirm what we do together with a lot of pride and praise” (H) “We praise him” (S) “We try a lot and encourage him” (C)	“Motivation plan” (H) “Encouragement” (S) “Give him tips and motivate him” (C)
	Applying a needs and age-oriented attitude (16 comments)	Parents are sensitive to children’s needs and treat them age-appropriately	x	x	x	“Listen carefully to the child: sometimes something works and sometimes it doesn’t. But we often don’t know why either” (H) “Adjustments according to his needs” (C)	“Make sure that information that might be of interest arrives and is read” (H) “I always remind him of his career aspirations so that he can find the strength and motivation for school” (S)
Accompanying, sharing, and supervising activities and participation (118 comments)	Accompanying child’s participation (41 comments)	Parents accompany their children on the way to school, at school, and while they participate in the community		x	x	“We accompany him to school, otherwise it wouldn’t work” (S) “If there is no teaching staff, I help out or the child stays at home” (S) “Mother always comes with him”(C)	“Sometimes bring her to school to relieve the stress of transferring in the morning so that she can bring all her energy to school” (S) “We support him by providing transport to the places, stay with him there and bring him home again” (C)
	Performing school activities at home (32 comments)	Parents accompany children in doing homework, repeating school tasks, or preparing for exams	x	x		“I stopped working … to be able to support him doing his homework better” (H) “We repeat the school lessons at home so that he progresses at school” (S) “Intensive support with homework” (S)	“Prepare for activities he will do at school (e.g. skiing or giving a lecture)” (S)
	Shared family activities (23 comments)	Parents expect their children to participate in family activities	x		x	“Joint activities of the family” (H) “Just take the child with you and include it, just like another child” (C)	“Nothing, he just goes along with it” (H) “Count on his participation” (C)
	Supervising (22 comments)	Parents organize care or supervision for their children	x	x		“Always make sure that someone is present” (H) “We applied for 100% teaching assistance” (S) “I stopped working … to be able to support him doing his homework better” (H)	“Check e.g. that he doesn't run from the bathroom to his computer” (H) “Involve external professionals, from school we do not expect a lot” (S)
Enhancing social relations and bridging (56 comments)	Building relationships with school personnel (30 comments)	Parents keep regular positive contact with teachers or other personnel at school to be informed and influence decisions		x		“Close collaboration with kindergarten teacher”(S) “Many talks with head of school and teachers, initiated through us” (S)	“Good contact with special needs teacher and main teacher” (S) “Regularly contact with school so we can support their decisions”(S)
	Reframing (26 comments)	Parents explain to the social environment (such as siblings, teachers, organizers, or parents of other children) the needs or behavior of their children	x	x	x	“We try to explain it to the little sister and thus bridge the gap between the two age groups” (H) “We speak openly with her teachers, explain her anxieties and try to find solutions together” (S) “To inform organizers and trainers in advance about possible complications or premature termination” (C)	“We are engaging in the relationship with parents of children our daughter is friends with” (H) “High transparency about diagnosis and problems with teachers” (S) “We try to inform children and parents about autism to increase their comprehension” (C)
	Inviting wider family and friends to participate (10 comments)	Parents maintain contact with wider family, friends, and parents of peers and invite them to participate with their children or visit them	x	x	x	“Using his sister as a motivator” (H) “We maintain good relationships with parents of peers” (S) “Invite a friend with kids or family member to participate with us” (C)	“Family cohesion” (C)
	Bringing in peers or other children (8 comments)	Parents invite or include peers or other children to support the participation of their child	x	x	x	“Invite the best friend of our daughter to come along” (H) “Bringing in his best friend who is a class above him” (S) “Emphasize that school friends are also there” (C)	“Laying a good foundation with peers from an early age (offering opportunities to play, cultivating relationships) so that the child experiences himself as an equal member and is more self-confident” (S) “Organizing with his friend play dates to make them more smooth” (C)
*Theme: “ACTIVITIES”*: parents influence participation through activities (219 comments: 104 children; 115 adolescents)							
Searching for and choosing interesting activities (58 comments)	Meeting interests of the child and conveying joy and fun (41 comments)	Parents address the interest of their children or make them attractive by conveying joy and fun. They also support implementing interests for participation	x	x	x	“He loves household experiments, cooking has a lot to do with physics” (H) “Refer to the things that are fun such as swimming in school, singing gymnastics” (S) “Interest-related activities (planetarium or museum)” (C)	“Include existing preferences and interests” (H) “Report on own experiences, how much fun they were” (S) “We encourage and support her to implement their own ideas, e.g. climate demonstration” (C)
	Giving them choices and asking for help (17 comments)	Parents offer choices or ask for help from their children to let them decide actively for their participation	x		x	“Let him choose the order of the tasks to be done” (H) “We make suggestions and let him choose” (C)	“We ask him for support” (H) “Give him opportunities” (C)
Planning and organizing activities and preparing the children and adolescents with ASD (127 comments)	Preparing children in detail beforehand (90 comments)	Parents prepare their children by informing them in advance (goal -oriented/repeatedly) and in great detail (precisely/instructively) about future participation. Additionally, parents of adolescents discuss a lot	x	x	x	“We discuss with him in advance possible difficulties that can occur during an activity and how one can then react to them” (H) “Short, clear instructions, no small talk, no questions, avoid filler words and focus on key communication” (H) “Ask carefully how the day was or otherwise get detailed information elsewhere and re-enact conflicts with Playmobil characters to prepare for the next school day” (S) “Provide enough information beforehand” (C)	“Early planning and information with multiple repetitions until the activity is carried out” (H) “A lot of discussions in advance” (S) “Precise preliminary information per activity who, how, when, what, where, how long, which goal” (S) “Convey security by preparing him for what to expect” (C) “Win argumentatively and create meaningfulness” (C)
	Planning and organizing (37 comments)	Parents plan activities beforehand and organize to benefit their children’s participation		x	x	“Pack the school bag” (S) “We arrange for our child organizational aspects” (C) “Endless search for hobby opportunities that fit the framework” (C)	“Checking the timetable, reminding when there are special events” (S) “We plan activities before we inform him” (C) “We pay for a painting course” (C) “Gather information about possible activities” (C)
Performing adapted activities (34 comments)	Starting, modelling, and grading (34 comments)	Parents start an activity and model for their children. They also grade the demands of activities	x		x	“Activities are graded, he takes part in sub-steps” (H) “Introduce the activity and observe him very closely to be aware of any questions or barriers” (C)	“Show and invite the child to involve himself” (H) “Again and again make offers for participation” (C)
*Theme: “TIME”* parents use aspects of time to support participation (70 comments: 34 children; 36 adolescents)							
Opting for regularity, more time and the right moment (70 comments)	Regularity and rituals (30 comments)	Parents stick to regularity and rituals to make daily tasks for their children more predictable	x	x	x	“We try to establish precise rules and rituals for as many activities as possible” (H) “Regularly daily routine” (S) “Try regularly” (C)	“Clear, non-negotiable minimum rules (getting up in the morning, showering, dinner together, handing over your mobile phone)” (H) “Regularly repeated events” (C)
	Creating and adapting time (25 comments)	Parents know that their children sometimes need more time and breaks in-between activities. Thus they use more time to reduce stress	x	x	x	“Take a break before some activities so that the child can adjust to it” (H) “A lot of recovery time at home” (S) “ Limit time (visits no longer than max 1–2 h depending on the activity” (S)	“Reduce the pace” (H) “Allowing time and avoiding pressure is usually successful, but it is difficult to get the child to school on time” (S) “…leave between every activity 1–2 h unplanned time” (C)
	Adapt to the state of mind at the moment (15 comments)	Parents are mindful about the right moment to address new topics or changes	x		x	“Introduction of the activity when the child is "on the receiving end", i.e. after being reasonably well-rested” (H)	“Wait for the right time” (H) “Align with his state of mind in the moment” (C)
*Theme: “OBJECTS”*: parents use objects to communicate and motivate (40 comments: children 17; adolescents 23)							
Providing visual communication and rewards (40 comments)	Visualizing (19 comments)	Parents communicate with graphical or visual methods	x	x	x	“Time timer” (H) “Pictograms such as weather app to understand weather conditions” (S) “Visualization with pictures or maps” (C)	“Partly detailed process plans that enable independent action” (H) “Prepare with maps or material from the internet” (S)
	Offering rewards (21 comments)	Parents offer rewards and incentives to acknowledge efforts and to motivate them to participate in unpleasant activities	x	x	x	“Promise the fulfillment of a greater wish and give points for successfully dealing with unpleasant things” (H) “Lure him with snails, his favorite animal, to motivate him to walk with us to school” (S)	“Offer of favorite food or favorite TV show” (H) “Incentives” (S) “"Horse trading" with rewards, e.g. media time” (C)
*Theme: “PLACE”*: parents chose places with just the right amount of stimuli (38 comments; children 26: adolescents 12)							
Choosing rooms to avoid or react to sensory overload (38 comments)	Organize possibilities for withdrawal and rest (20 comments)	Provide opportunities for withdrawal or rest to avoid sensory overload	x		x	“Provide relaxation and opportunities for withdrawal at home, to allow his adaptation capacity, which is overstretched through school, to be balanced again.” (S) “Codeword "car" or "home" when it gets too much” (C)	“Adjust the furniture (hammock for reading, works very well)” (H) “We stop the activities early to avoid overload” (C)
	Choosing rooms and furniture based on sensory aspects (18 comments)	Parents adapt rooms and furniture to the needs of their children or teach them coping strategies	x	x	x	“Learn strategies to cope with sensory overload” (H) “Organize edge seating, window seat” (C)	“Avoid bad-smelling situations” (H) “Familiar surroundings are important. We only go shopping at certain supermarkets, as there are fewer attractions there than at others. According to our son, these discount stores are structured logically and are clearer” (C)

In the following paragraph, the highest percentages of “supports” and “barriers” are reported along each of the three settings. At home, parents judged most of the environmental aspects as “supports.” Specifically, “resources” (such as *“supplies,” “information,” “money”)* were rated highly. “*Sensory quality”* and *“social demands of activities”* were perceived as strong “barriers” in the home setting. At school, *“attitudes”* of teachers and staff and most of the “resources” (such as “*programs and services,” “information,”* and *“time”)* were rated as strong “supports.” Highly endorsed “barriers” at school were the “*sensory quality”* and the *“social demands of activities.”* In the community, parents judged that only *“safety”* was a clear support regarding aspects of “helpfulness,” while most “resources” (such as “*transportation*,” “*policies and procedures*,” “*information*,” “*time*,” and “*money”)* were also rated as strong “supports.” Clear “barriers” in the community were “*sensory quality*” and the “*social demand of activities.”* The community was the setting in which most parents chose to swing between *“sometimes helps and sometimes makes harder.”*

**Fig. 1:**
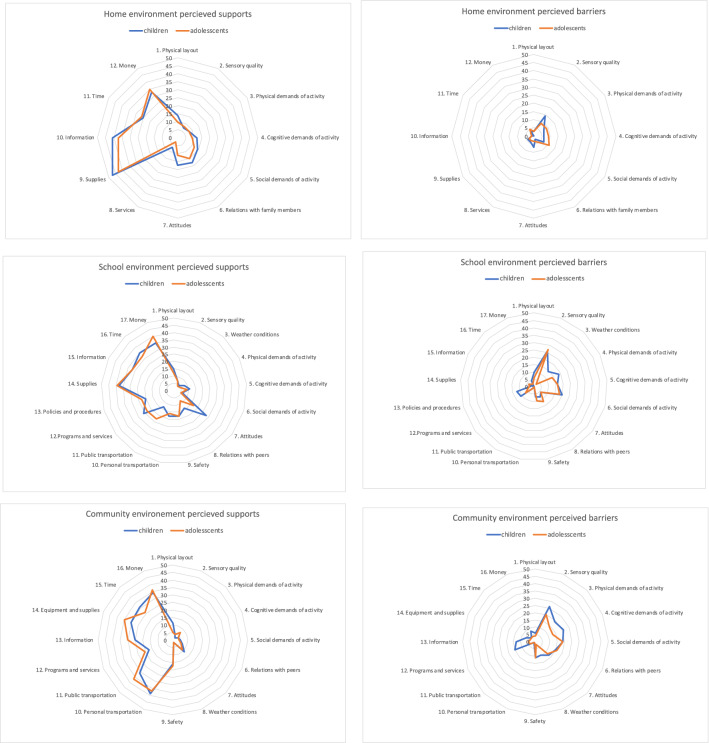
Percentages of supports and barriers in two age groups as perceived by parents

A summary of “supports” and “barriers is presented for each setting graphically in Fig. 1. It is apparent (as reported earlier) that in all three settings, parents reported more “supports” than “barriers,” with a decline in support from home to school to the community. Next, the distribution regarding the 45 environmental aspects remained nearly similar for both age groups. Differences over 10% between age groups were found in “*weather conditions,”* which were stronger “barriers” at home in younger ages. Supportive “*attitudes*” at school were higher in children with ASD than in adolescents with ASD, while the reverse was true for “*public transport*” at school. Not all “supports” and “barriers” per setting were equally distributed. Parents in all three settings perceived more “supports” in aspects summarized as “resources” (such as “*services*,” “*information*,” “*time*,” “*money*”) than those summarized under “helpfulness” (such as “*physical layout,”* “*sensory qualities,”* “*physical demands*,” “*social demands*,” “*attitudes,”* or “*social relations”).* This is mirrored by “barriers” in all settings: parents perceived more “barriers” in aspects summarized under “helpfulness” than those summarized as “resources.”

### Contextual Strategies of Parents to Support Participation

The following paragraph contains a summary of contextual strategies using the five themes of “people,” “activity,” “time,” “objects,” and “place,” which are presented in nine clusters such as “encouraging participation” or “accompanying, sharing, or supervising activities.” Each cluster contains several single strategies. Table 5 lists five themes and nine clusters and describes 22 single strategies, for which exemplary quotes from three settings and two age groups are displayed. Most strategies were used in all three settings. In total, 624 strategies were mentioned (home: 231 strategies; school: 220 strategies; community 172 strategies). Only minor differences between the age groups were found. The most mentioned contextual strategies are presented first.

### “People”: Parents Use their Relationships to Support Participation

Under “people”, 41% (*n* = 257 strategies) of all data are summarized in three clusters of strategies.

In the first cluster, called *encouraging participation*, parents used their empathic relationships with their children to motivate them, appraise their efforts, and encourage involvement. Being observant and sensitive to their children’s needs allowed parents to react immediately and adapt their appraisal and their strategies.

In the second cluster of strategies, parents *accompanied their children’s participation* in various ways. Accompanying transitions to school was often mentioned. Shared participation in the community was described as securing and affirming for children and adolescents with ASD. In the home setting, parents described the meaningfulness of shared family activities and how they naturally expected participation and involved their children with ASD in these activities. Performing school activities together with their children at home was another accompanying strategy. Parents supported their children’s learning and prepared them to cope with school demands. Finally, in the home setting, parents supervised their children’s participation or organized other persons to supervise them.

In the third cluster of strategies, parents *enhanced the social relationships* of their children as well as their own; they actively worked to maintain a positive relationship with school personnel to influence school activities and become informed about their children’s issues at school (15% of all strategies at school). To enable a positive participation experience, parents reframed (such as explained and briefed) the social environments regarding the needs and behavior of their children. This enhanced understanding, empathy, and positive attitudes for their children with ASD. Involving wider family, friends, children, and peers was mentioned more often in home and community settings. Only 8 comments mentioned involving other peers actively.

### “Activities”: Parents Influence Participation Through Activities

Under “activities”, 35% (*n* = 219) of all comments were summarized in three clusters:

The first cluster of strategies was based on parents’ *intensive search for suitable activities* for their children. When choosing these activities, they considered their children’s interests, conveyed joy and fun to them, and motivated them by providing choices or asking them for help. Specifically for community participation, parents reported difficulties finding optimal settings and activities for their children with ASD.

In the second cluster of strategies, parents *planned and organized these activities* in detail to be able to accurately prepare their children with ASD for later activities. 15% of all comments referred to preparing children and adolescents with ASD beforehand. Parents mentioned how they repeatedly provided goal-oriented information about future activities. To ensure a sense of security, parents reported delivering the information in a precise and instructive manner.

In the third cluster under “activities,” parents *started, modelled, graded, and adapted the demands of activities,* so their children with ASD could join and have a positive participation experience (as the ultimate goal).

The next three parental strategies are clustered in one cluster with similar strategies.

### “Time”: Parents Use Aspects of Time to Support Participation

Applying strategies connected to “time” (11% of all comments), parents opted for regularity and rituals (often also in combination with rules), provided altogether more time for participation experiences, and reduced the pace of activities. Parents mentioned they reduced the total length of activities or planned free time slots between activities. Parents described further how they were mindful of the actual state of mind of their child. Waiting for the “right” moment to introduce a new activity or being well-rested before a socially-demanding participation experience seemed central.

### “Objects”: Parents Use Objects to Communicate and Motivate

Parental strategies around “objects” (6% of all comments) were connected to the availability of materials to visualize concepts as this was described as easing communication. “Objects” were also mentioned in combination with rewards and incentives used to motivate the children and adolescents with ASD for externally demanded activities that were often necessary but unpleasant, such as showering or joining a social event.

### “Places”: Parents Choose Places with Just the Right Amount of Stimuli

Last, parental strategies around “places” (6% of all comments) described themes connected to sensory overload. Parents chose rooms or spaces to prevent sensory overload or taught their children strategies to prevent it. Next, parents applied strategies to react to possible sensory overload by looking for places to withdraw or by providing opportunities to rest and restore from sensory overload.

## Discussion

The purpose of this study was to explore parental perspectives on the environmental supports and barriers to the participation of children and adolescents with ASD in Switzerland, as well as related contextual strategies.

Answers to the first question showed that one half of parents perceived more “supports” than “barriers” in all three settings. “Supports” and “barriers” were qualitatively different, but “*sensory aspects”* and “*social demands of activities*” were rated as the highest “barriers” in all three settings. The remaining half of parents swung between more supports and more barriers and thus did not perceive a general “barrier” or “support” in the environment.

Results for the second question showed that contextual strategies were overly connected to “people” (41%) (and thus were social in nature) and “activities” (36%) (representing activity changes to support participation). Most parental strategies were reported similarly in all settings and in both age groups, indicating that environments may stay the same during childhood and adolescence.

### Differences Between Supports and Barriers

This study resented “supports” and “barriers” descriptively and graphically without summary scores. This in contrast to other studies, which used summary scores of supportiveness (Bakaniene & Prasauskiene, 2020; Kaelin et al., 2020) or combined the answers “not an issue” and “usually helps” (Egilson et al., 2018). Without summary scores it became obvious that in the present study “supports” were twice as numerous as “barriers,” and were at the same time different in nature. “Supports” (such as “*services,*” “*information,*” “*time,*” “*money,*” *“equipment and supplies,*” and *“transportation”)* are either automatically provided by the Swiss social system or families in Switzerland can opt for them. Families can opt, for example, to reduce parental workload and thus gain additional time to support the participation of the child or adolescent with ASD.

This freedom of choice contrasts with the perceived “barriers” (such as *“sensory quality” “cognitive demands of activities,” “social demands of activities,” “relationships,”* and *“attitudes”)* which are part of the built or socially-constructed environment and go beyond the scope of single families. By scoring these things as “barriers,” parents expressed a certain helplessness. Their ability as parents or families to influence things like environmental noise, social rules, or attitudes to accommodate the needs of their children with ASD is perceived as rather low (Butler & Gillis, 2011; Jones & Harwood, 2009). Instead, these areas contain potential for service providers and society in general to support parents with children with ASD effectively. Examples for this are general noice restricting constructions or regulations, a higher diversity regarding fulfilling unspoken social rules and campains to address negative stereotyping of persons with autism.

Half of the parents swung between reporting the environment as a clear “support” or “barrier,” but instead said the environment served *“sometimes as a help* and *sometimes as a hindrance*” or was “*not an issue.*” Eglison et al. (2018) reported a similar pattern. The authors see two possible explanations for this uncertainty: first, parents found it hard to determine an overall influence of the environment, as demanded by the construct underlying PEM-CY (Krieger et al., 2020b). A dichotomous answer option might not be adequate to capture the complexities of environmental influence. Second, environmental aspects are dependent on enacted contextual situations as proposed by King et al (2018). For example, whether “*being together with other people*” is supporting or hindering in a setting depends on the persons in the setting. In a school setting, a librarian might be supportive of a child with ASD, while in the same setting, a sports teacher may not be. This swing was primarily found in the community setting. The authors suspect this is because community or public environments are more diverse and less predictable.

### Supports and Barriers in Specific Settings

“Supports” and “barriers” did not differ considerably across the three settings in our sample, a finding similar to that seen in children and adolescents with non-ASD disabilities (Bakaniene & Prasauskiene, 2020; Coster et al., 2011; Shabat et al., 2021). However, it is pointed to particularities at the home and the community setting:

In a home setting, parents have the operating agency to adapt the environment to the needs of their children and adolescents with ASD. Parents rated *“the relationships with family members*” as the strongest “support” in the home. Family relationships were found to be highly important in other research as well (Krieger et al., 2018; Orsmond et al., 2006; Taylor & Seltzer, 2011). Parents rated *“sensory quality”* as the highest “barrier,” indicating a need to find suitable solutions (Hazen et al., 2014; Little et al., 2014). An intervention framework to address sensory issues in the home exists to guide service providers and health workers (Ashburner et al., 2014). It proposes universal design principles and self-regulation strategies to optimize participation experiences for children and adolescents with ASD.

In community settings, the reported barriers from Switzerland (CH) exceed those reported from Australia (AU) (Devenish et al., 2020). Differences were specifically found in “*relationships with peers*” (CH 31%; AU 8%), “*safety*” (CH 22%; AU 8%), “*sensory quality*” (CH 45%; AU 33%), and “*social demands*” (CH 40%; AU 35%). The age difference between these two samples (CH: 5–17 years; AU: 5–12 years) could be one explanation for the discrepancy. However, the authors assume that attitudinal, cultural, or policy differences between the two countries contribute the most to these differences. Unfortunately, most research into attitudes and stigmatization in ASD is nationally-based (Thompson-Hodgetts et al., 2020). From an anthropological viewpoint, research suggests that cultural differences can even mediate environmental differences such as maternal education, ethnicity, and the perceived negative impact of ASD (Carr & Lord, 2012). Region-based cultural aspects need to be included in any further research focusing on transnational environments. Further, a supportive environment should be perceived as supportive from the persons with ASD themselves (Gardiner & Iarocci, 2014).

### Contextual Parental Strategies a Combination of “People” and “Activities”

The most common contextual strategies of parents (“people” and “activities”) correspond to the two main diagnostic features of ASD (American Psychiatric Association, 2013): (1) social-communicative differences, connected to strategies that were condensed into “people” and (2) restricted and repetitive behavior, connected to strategies that were condensed into “activities”. As such, parents provide support targeted to address autistic traits that hinder the participation of their children with ASD. Further, the results generally reflect the seven strategic patterns Egilson et al. (2018) found in adolescents with and without ASD, but this present study tailored them more specifically to children and adolescents on the autistic spectrum.

In the contextual strategies labeled “people,” parents use their confidential relationship with their children and adolescents with ASD to motivate, provide company, and serve as gatekeepers for new social contacts and participation. Parents and families are the most critical environment in childhood (Rosenbaum & Gorter, 2012). While less dependence on parents is assumed in adolescence (Imms et al., 2017), parents are still extensively involved in the lives of adolescents with ASD. The constant work of securing and helping to form connections (Krieger et al., 2018), which can involve the parent taking on many different roles—such as motivator, door-opener, or companionship—to create a supportive environment for children and adolescents with ASD, can be tiring over time. Other persons from the wider support circle—friends, practitioners, or service providers—can relieve some of the strains parents experience. For example, school personnel can replace parents in supervising homework, or other children can walk together and socialize with youth with ASD (Ziviani et al., 2006). Hence, children and adolescents need company and consistency, but these things do not necessarily have to be provided by parents (Adams et al., 2019; Krieger et al., 2018, 2020a). Although parents often see themselves as gatekeepers in connecting their children with others, it is noticeable how few strategies parents recalled using to connect their children with peers. Service providers may focus on educating parents about how to support peer-to-peer interaction. Shared activities, such as walking together to school or working on homework in a group, increase social participation in children and adolescents with ASD (Chen et al., 2016).

“Activities” comprised nearly a third of all parental strategies, indicating how frequently parents search for and adapt activities and motivate and inform their children about them. The poor executive functioning combined with less flexibility and poor problem-solving capacities seen in ASD might be one reason for this (Lopez et al., 2005). From all parental strategies, 15 percent comprised informing and making children and adolescents knowledgeable about a topic. Adolescents with ASD expressed a high need to get information and become knowledgeable before they could participate in a community (Krieger et al., 2018, 2020a), something that goes beyond parental strategies and can inform service providers and therapists as well (Adams et al., 2019).

Three strengths of this study are notable. First, it uniquely presents estimates of 45 activities regarding the participation of children and adolescents with ASD in three settings in Switzerland. Second, it describes for the first time parental contextual strategies tailored to children and adolescents with ASD which cover the whole phenotype of autism. Despite the wide use of the PEM-CY with various populations and research questions, parental strategies are rarely published, probably due to limited space in journals. Our paper’s last strength lies in the combined use of contextual theory (King et al, 2018) with parental strategies, which makes the transactional interconnectedness between a person and contexts, setting, and environment for the sake of an enacted participation more transparent.

In addition to a first qualitative analysis, the authors found it useful to apply the context definition of King et al. (2013, 2018) for our second analysis. While doing so, it was noticed that attitudes of others, institutional barriers, or social-economic situations were not reflected in the definition of contexts. These were only mentioned incidentally and did not interfere with the coding of parental strategies.

Several limitations of this study require consideration when interpreting the findings. Due to the population-based nature of the sample and the fact, that participants identified themselves as parents of children or adolescents with ASD, a participant bias might be possible. Next, as for all parent reports, the possibility of under- or over-rating is inherent (Allonsius et al., 2021; Rios & Scharoun Benson, 2020). Parents needed to score an environment they are fundamentally connected with. A selection bias is possible because filling in the PEM-CY is challenging and often missing data sets are described (Chien et al., 2020). After consenting, the enrollment rate was 85% and a completion rate 73%, indicating a lost a quarter of participants after reading the instructions and while completing the PEM-CY. No particular pattern of those who quit was observed, but the sample of the study represent a higher education rate as reported from official statistics (Bundesamt für Statistik, 2021). Regarding family constellation, workload and the number of spoken languages in the family, the sample of this study data fits those from official statistics (Bundesamt für Statistik, 2017). Further, another demographic aspect in combination with the environment was noteworthy: 74% of the Swiss population lives in urban or suburban communities (Bundesamt für Statistik, 2021). Instead, in the sample of the study, only 42% of participants were urban and suburban residents, while 57% of participating families were living in rural communities. It is yet unclear whether this is a possible self selection bias, or whether parents with a child with ASD chose due to aspects such as availability, financial, or social reasons rural environments. Fourth, the authors acknowledge that Covid-19 has changed the face of society, accessibility, and services due to the long ripple effects of the pandemic. The authors could not have foreseen nor controlles these effects, which might influence the interpretation of the results of this present study. Finally, as inherent in any cross-sectional study design, causality cannot be attributed and a direct connection between the two age groups cannot be supposed. However, the authors deliberately chose a purely descriptive analysis, and the results should be informative to health care providers and policy developers not acquainted with the PEM-CY construct. Due to the nature of a cross-sectional study, qualitative and quantitative methodology may not equally be presented.

## Conclusion and Further Research

The present study provides a diverse and in-depth look into environmental and contextual aspects for the participation of children and adolescents with ASD as seen from parents’ point of view. Half of parents of this study from Switzerland saw more supports than barriers for their children’s participation. However, barriers such as attitudes, social demands, or sensory qualities of the environment were described as less tangible and less changeable for parents. Parental perspectives on participation and their contextual strategies need to be considered in environment-based interventions to support the social participation of children and adolescents with ASD. This can simultaneously reduce caretaking-related strain in parents of children and adolescents with ASD.

More research with this transactional perspective on the interconnectedness between environment, context, and participation of youth with ASD is needed; environment-based interventions to increase their participation are also important.

## Supplementary Information

Below is the link to the electronic supplementary material.

Supplementary file1 (DOCX 240 kb)
