# Supplementary material for: Parents’ Perceptions: Environments and the Contextual Strategies of Parents to Support the Participation of Children and Adolescents with Autism Spectrum Disorder—A Descriptive Population-Based Study from Switzerland
Source: J Autism Dev Disord. 2022 Dec 20;54(3):871–93. doi: 10.1007/s10803-022-05826-2 (PMC9765345; doi:10.1007/s10803-022-05826-2)
Supplement: Supplementary file 1 — Supplementary file1 (DOCX 240 kb) [file 10803_2022_5826_MOESM1_ESM.docx]

**ESM 1 Online Resource 1:** Flowchart Selection

**ESM 2: Online Resource 2**: Parents rated manifestation of ASD during the last 4 months^1^

|  | **Children with ASD**  *age 5-11*  *n=66* | | **Adolescents with ASD**  *age 12-17*  *n=55* | | **Total number of youth**  *age 5-17*  *n= 115* | |
| --- | --- | --- | --- | --- | --- | --- |
|  | median | IQR | median | IQR | median | IQR |
| 1. Ability of expressive language | 5 | 3.5-6 | 6 | 5-6 | 6 | 4-6 |
| 1. Use of communication aids | 6 | 5-6 | 6 | 6-6 | 6 | 5-6 |
| 1. Intellectual abilities | 6 | 5-6 | 6 | 4-6 | 6 | 4-6 |
| 1. Self-harming behavior | 6 | 4.5-6 | 6 | 5-6 | 6 | 5-6 |
| 1. Reaction to changes | 3 | 1-4 | 3 | 1-4 | 3 | 1-4 |
| 1. Repetitive behavior | 4 | 2-5 | 4 | 3-5 | 4 | 2-5 |
| 1. Restricted behavior | 4 | 3-5 | 4 | 2-5 | 4 | 3-5 |
| 1. Selective eating | 4 | 2-5 | 4 | 3-6 | 4 | 2-5 |
| 1. Sleeping situation | 3 | 2-5 | 4 | 3-6 | 4 | 2-5 |
| 1. Interaction with other children | 3 | 3-4 | 4 | 2-4 | 4 | 2-4 |
| 1. Age appropriate independence | 3 | 2-4 | 4 | 2-5 | 3 | 2-4 |

^1^highest number (6) indicates not been affected, lowest number (1) indicates being strongly affected as rated by parents

Calculations based on Tukey’s range tests
